# Niagara University—Medical Department

**Published:** 1890-04

**Authors:** 


### NIAGARA UNIVERSITY—MEDICAL DEPARTMENT.

THE present college term in this institution will cease April 15th. The examination of students by the Board of Examiners for the degree of Doctor of Medicine, will take place on that day at 10 A. M. The commencement exercises will be held at the Star Theatre in the evening. Dr. L. S. McMurtry, of Louisville, Ky., whose reputation as a gynecologist extends far beyond the confines of this continent, will deliver the address to the Alumni, and Rev. Henry A. Adams, the popular and eloquent rector of St. Paul's Church, in this city, will give the address to the graduates.

The Alumni Association will meet on the same day, the morning session being held at the College on Ellicott street. Prof. Herbert Mickle will give the address of welcome, and Dr. Geo. W. T. Lewis the President's address. At the Y. M. C. A. Building, on West

Mohawk street, the afternoon session will be held, and papers of great interest and value will be read by Surgeon William S. Tremaine, U.S.A., Emeritus Professor of Surgery, entitled, Remarks on the Treatment of Fibroid Tumors of the Uterus; Dr. Joseph Hoffman, of Philadelphia, on The Treatment of Lesions at and about the Head of the Colon; and Dr. Burt G. Wilder on The Sub-frontal Gyre (Broca's Convolution) in Man and Apes.

The Alumni banquet will follow at the conclusion of the graduating exercises.

The Spring course of lectures will open on Monday, April 21st, and continue four weeks.

